# Text-message Reminders in Colorectal Cancer Screening (TRICCS): a randomised controlled trial

**DOI:** 10.1038/bjc.2017.117

**Published:** 2017-04-25

**Authors:** Yasemin Hirst, Hanna Skrobanski, Robert S Kerrison, Lindsay C Kobayashi, Nicholas Counsell, Natasha Djedovic, Josephine Ruwende, Mark Stewart, Christian von Wagner

**Affiliations:** 1 The Research Department of Behavioural Science and Health, University College London, Gower Street, London WC1E 7BT, UK; 2 Center for Population and Development Studies, Harvard T. H. Chan School of Public Health, Harvard University, Cambridge, MA 02138, USA; 3 Cancer Research UK & UCL Cancer Trials Centre, Cancer Institute, University College London, 90 Tottenham Court Road, London W1T 4TJ, UK; 4 St Marks Bowel Cancer Screening Centre, St Marks Hospital, Watford Road, Harrow, Middlesex HA1 3UJ, UK; 5 NHS England London Region, Southside, 105 Victoria Street, London SW1E 6QT, UK

**Keywords:** colorectal, cancer screening, randomised controlled trial, reminder, uptake, text-message

## Abstract

**Background:**

We investigated the effectiveness of a text-message reminder to improve uptake of the English Bowel Cancer Screening programme in London.

**Methods:**

We performed a randomised controlled trial across 141 general practices in London. Eight thousand two hundred sixty-nine screening-eligible adults (aged 60–74 years) were randomised in a 1 : 1 ratio to receive either a text-message reminder (*n*=4134) or no text-message reminder (*n*=4135) if they had not returned their faecal occult blood test kit within 8 weeks of initial invitation. The primary outcome was the proportion of adults returning a test kit at the end of an 18-week screening episode (intention-to-treat analysis). A subgroup analysis was conducted for individuals receiving an invitation for the first time.

**Results:**

Uptake was 39.9% in the control group and 40.5% in the intervention group. Uptake did not differ significantly between groups for the whole study population of older adults (adjusted odds ratio (OR) 1.03, 95% confidence interval (CI) 0.94–1.12; *P*=0.56) but did vary between the groups for first-time invitees (uptake was 34.9% in the control and 40.5% in the intervention; adjusted OR 1.29, 95% CI 1.04–1.58; *P*=0.02).

**Conclusions:**

Although text-message reminders did not significantly increase uptake of the overall population, the improvement among first-time invitees is encouraging.

Colorectal cancer (CRC) is the fourth most common cancer and the second leading cause of cancer death in the United Kingdom (Cancer Research UK, 2016a, b). In England, the National Health Service (NHS) runs the Bowel Cancer Screening Programme (BCSP), an organised CRC screening programme that offers biennial guaiac faecal occult blood testing (gFOBt) to men and women aged 60–74 years. Screening is widely recommended for the early detection of CRC and repeated participation in CRC screening has been shown to reduce CRC mortality by up to 25% (Scholefield *et al*, 2002; Hewitson *et al*, 2008; Levin *et al*, 2008; Sung *et al*, 2015). However, overall uptake of CRC screening in England is low at approximately 56% (House of Commons, 2014).

Commonly reported reasons for non-participation in CRC screening include forgetting or not getting around to completing the test kit and being too busy (Janz *et al*, 2007; van Rijn *et al*, 2008; van Dam *et al*, 2013; Lo *et al*, 2015b). Previous studies have shown that various modalities of reminders are effective at improving CRC screening uptake, including telephone and postal reminders (Baron *et al*, 2008; Power *et al*, 2009; Camilloni *et al*, 2013). Telephone reminders have been found to be the most effective reminder modality, improving CRC screening uptake by up to 21% (Myers *et al*, 2007; Segnan *et al*, 2010; Camilloni *et al*, 2013; Davis *et al*, 2014). However, the cost of telephone reminders is often prohibitive in the context of publicly funded screening programmes due to the additional labour they require (Segnan *et al*, 2010).

In the United States, a randomised controlled trial (RCT) examining the effectiveness of a multicomponent strategy to increase uptake of gFOBt through community health centres found that text-messages (also referred to as Short Messaging Service), when used in conjunction with postal and automated telephone reminders, achieved uptake of 82%, compared with 37% in the usual care group (Baker *et al*, 2014). In the context of organised screening in the United Kingdom, two recent studies found that preappointment text-message reminders were effective at increasing attendance at routine breast screening appointments (Icheku and Arowobusoye, 2015; Kerrison *et al*, 2015). The effectiveness of text-message reminders to promote uptake of gFOBt in the NHS BCSP, however, has not been examined (Senore *et al*, 2015). Unlike preappointment reminders for the NHS Breast Screening Programme, a reminder for CRC screening in the BCSP would act as an additional prompt to complete and return a gFOBt kit beyond the standard 4-week postal reminder (Halloran, 2009).

Text-messages are often considered to be the next best alternative to telephone reminders, as they have the advantage of providing instant and direct transmission at a low cost (Gurol–Urganci *et al*, 2013). They also have an advantage over postal reminders, as the direct transmission of text-messages to a person’s mobile phone means they are unlikely to be misplaced as items sent through the post (Kaplan, 2006). The effectiveness of delivering text-messages is likely to be influenced by mobile phone coverage and the accuracy of mobile phone records in the clinical systems. Mobile technology has the potential to provide outreach and access to people regardless of socioeconomic status, race, ethnicity, or location, with 93% of the UK population personally owning or using a mobile phone (Ofcom, 2016). However, challenges with integrating mobile technology with primary care remain, as a recent study reported that only 39.8% of the eligible population had a registered mobile on their GP’s clinical system (Kerrison *et al*, 2015).

The effect of a text-message reminder for CRC screening is likely to be different based on an individual’s history with the screening programme, specifically whether they are first-time or repeat invitees in a given screening round. Uptake at first-time invitation is the strongest predictor of repeat uptake in the second round of invitations. A recent population-based study showed that uptake of the second round of invitations was 87% among people who previously participated in the first round (Lo *et al*, 2015a). In comparison, uptake among people invited for CRC screening for the first time is only around 54% (von Wagner *et al*, 2011). Furthermore, a recent series of trials piloting interventions to improve uptake of the BCSP in England found that interventions had a significantly stronger impact among first-time invitees compared with those who had previously been invited (White *et al*, 2015). Thus there is greater opportunity to promote uptake among first-time invitees, which would lead to greater future adherence among screening-eligible adults.

The primary aim of this RCT was to test the effectiveness of a text-message reminder to increase gFOBt uptake. The outcome measure was the total proportion of invitees adequately screened 18 weeks after the initial invitation was sent. Our secondary aims were to examine whether or not a text-message reminder is more effective in improving uptake among first-time invitees than repeat invitees and to establish the efficacy of the text-messages by testing the effectiveness among the non-responders who also had a registered mobile number. The findings will be important to show the impact of the intervention on the overall population and also the subgroups who are of interest of the text-message reminder intervention.

## Methods and materials

### Study design and setting

We performed a two-arm RCT in London across six Clinical Commissioning Groups ((CCGs): NHS organisations that manage patient care in GPs in defined geographical areas): Croydon, Greenwich, Hammersmith and Fulham, Hounslow, Lewisham, and West London. The study was carried out in collaboration with the NHS Bowel Cancer Screening Hub in London (hereafter referred to as ‘the hub’), who were responsible for creating and maintaining the study database, and iPlato (iPlato, 2013), who were responsible for randomising eligible adults and delivering the text-message reminders. iPlato is an NHS Information Governance approved mobile health (m-health) organization, which provides patient care messaging services to primary and secondary health care in England.

From age 60 and up to age 74 years, the NHS BCSP biennially invites all men and women take part in CRC screening using the gFOBt kit. The gFOBt pathway includes a baseline invitation to initiate a screening episode, which is characterised as an 18-week-long period for the adequate completion of a gFOBt kit. A week after the initial invite, the kit is dispatched, and a reminder is sent out on the fifth week of the initial invite if the kit had not been returned. Unless the results are abnormal, CRC screening does not include further investigations beyond the gFOBt pathway.

**Figure 1:**
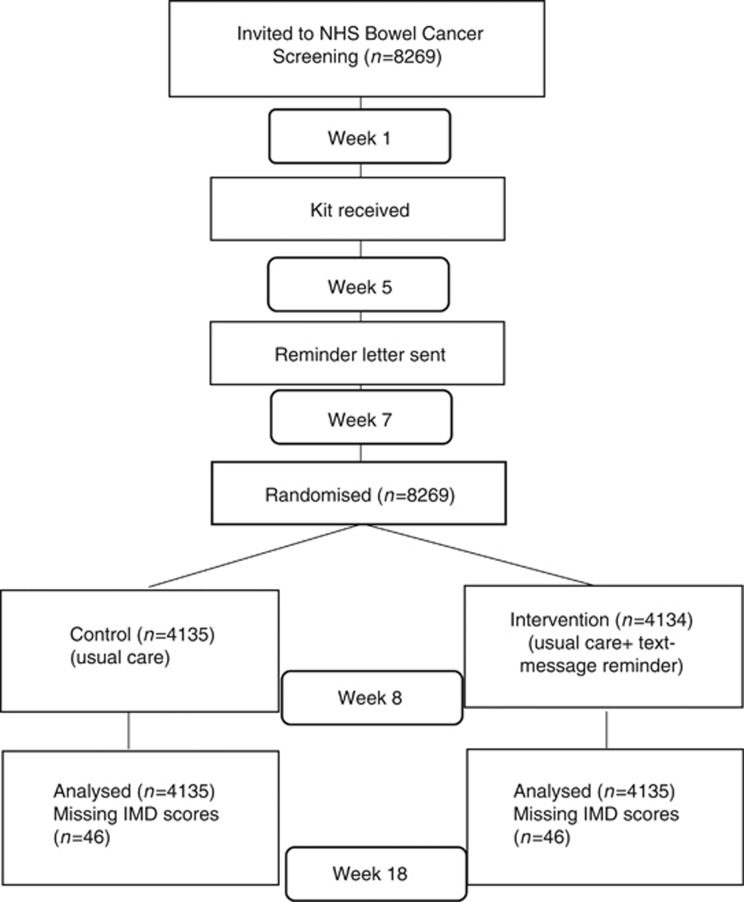
**Study consort diagram.**

In this two-arm trial, individuals who were randomly allocated to the control group were invited to take part in the BCSP as per the usual care pathway, while individuals randomly allocated to the intervention group were additionally sent a text-message reminder if they had not returned their test kit 7 weeks into their screening episode and had a mobile number stored on their GP’s clinical system (see Figure 1).

Further details of the intervention design and procedures for this RCT are available in the published protocol (Hirst *et al*, 2016).

### GP recruitment

All GPs situated within the six CCGs were emailed and invited to participate in this study between November and December 2015. This invitation was followed by weekly email reminders. Practices were eligible if they already had an existing patient messaging service, which ensured patient consent for text-messages. In order to facilitate automated text-messages, all practices had to connect to the study-specific iPlato server, irrespective of their routine m-health provider in use for text-messages. In total, 144 out of 295 practices (48.8%) agreed to take part and returned their consent forms. Owing to technical difficulties, only 141 out of 144 GPs were successfully connected to iPlato. Low response rate was considered to be an outcome of the eligibility criteria (Hirst *et al*, 2016).

### Trial procedures

#### Identification

Between January and March 2016, the hub retrospectively identified all men and women aged 60–74 years from all 141 practices to be included in the study based on the start date of their gFOBt screening episode. Each week, everyone who had been invited 7 weeks back were included, irrespective of whether or not they had already completed and returned their faecal occult blood test kit.

The Hub then transferred the list of the screening adults to iPlato. The weekly file contained relevant data to the individuals’ screening statuses, including their unique identifier used within the NHS, referred to as the ‘NHS Number’, the kit return status (Yes/No), the date of screening invitation (Date/Month/Year) used to calculate the date of a text-reminder, and GP code for tailored text-messages (a unique GP identifier).

#### Data processing and generation

On receipt of the data at iPlato, individuals were randomised each week in a 1 : 1 ratio to either the intervention or control condition. Randomisation was conducted using Mersenne Twister: a computerised, pseudorandom number generator (Matsumoto and Nishimura, 1998).

After randomisation, registered mobile number status (Yes/No) was added to the weekly data file, as well as delivery status of the text-message reminder (Yes/No) if the person was in the intervention group and eligible to receive a text-message. The last text-message reminder was sent on the 18 March 2016. When the last of the individual screening episodes were closed 18 weeks after the delivery of the baseline FOBt invitation, a complete data set was returned via secure email transfer to the Hub.

#### Data merge and anonymisation

The Hub made a record of whether or not a gFOBt had been adequately completed by the individual within the study database and then added individual-level data on age, gender, the name of the CCG, the screening episode sequence number (i.e., the number of screening test kits received by the eligible individual), and area-level of deprivation (i.e., derived from the ‘Index of Multiple Deprivation’ (IMD) score) to the data set. The IMD uses census-derived indicators of income, education, employment, living environment, health and disability, barriers to housing and services, and crime at small-area level to generate a scale from 0 (least deprived) to 80 (most deprived). For the purposes of our analysis, IMD scores were categorised into quintiles of the national distribution to enable comparisons between individuals living in the most and least deprived groups of areas to be made. Prior to transferring to the research team for analysis, the Hub additionally removed identifiable data (e.g., NHS number, GP code, and postcode), which had been used to facilitate the research.

### Trial registration and ethics

The study was approved by the East Midlands National Research Ethics Service (15/EM/0159) and is registered with the International Standard Randomised Controlled Trial Number (ISRCTN) registry for transparency (ISRCTN70904476 (18/09/2015)). The study has also been reviewed by the Confidentiality Advisory Group (CAG) and granted full approval (15/CAG/0156), permitting iPlato to process identifiable information for the purposes of this study to be able to send automated text-messages from participating GPs.

### Statistical analysis

The characteristics of the screening-eligible population were demonstrated using descriptive statistics. The primary study outcome, the proportion of invitees who returned a test kit within 18 weeks, was assessed on an intention-to-treat basis. A subgroup analysis using interaction terms were included to test the intervention by invitation status (first-time invitee=0; repeat invitee=1). The invitation status variable was computed by dichotomising the screening episode sequence number. A secondary analysis was also performed, restricting the sample to individuals who had not returned their kit within 8 weeks and had a registered mobile number on their GP’s clinical system. Analyses were conducted using multivariable logistic regression models adjusting for sociodemographic factors (age bands, gender, IMD quintiles, and CCG). Univariable logistic regression results were reported (Supplementary Table S7). An exploratory analysis was conducted to identify the predictors of having a registered mobile number. All results are presented using odds ratios (ORs), 95% confidence intervals (CIs), and *P*-values. *P*-values <0.05 were considered as statistically significant.

## Results

### Population characteristics

In total, 8269 men and women were eligible for CRC screening and subsequently invited to the NHS BCSP during the study identification period. Of these, 4135 were randomised to the control group and 4134 were randomised to the intervention group. Baseline characteristics are shown in Table 1: Individuals invited to screening had a median age of 66 (range 65–69) years, just over half were women (52.0%), and most had been invited to the NHS BCSP more than once (81.4%). Less than one-tenth were from the least deprived IMD quintile (8.6%), and about one-fifth were from the most deprived IMD quintile (21.1%). The proportion of invitees was highest in Croydon (21.7%), followed by Greenwich (20.7%), Hounslow (19.9%), Lewisham (16.3%), and West London (14.5%) and lowest in Hammersmith and Fulham (7.0%).

### The impact of the intervention

Overall, 40.2% of individuals were adequately screened; there was no significant difference in the proportion adequately screened between the intervention (40.5%) and control groups (39.9% OR=1.03; 95% CI: 0.94–1.12; *P*=0.56) (Table 2).

### Factors associated with CRC screening uptake

CRC screening uptake was lower among men than women (37.4% *vs* 42.8% OR=0.80; 95% CI: 0.73–0.88; *P*=0.001), higher among people aged 70–74 years than those aged 60–64 years (38.2% *vs* 42.8% OR=1.26; 95% CI: 1.11–1.43; *P*<0.001), and lower among the most deprived IMD quintile *vs* least deprived quintile (34.0% *vs* 51.5% OR=0.53; 95% CI: 0.44–0.64; *P*<0.001). Uptake also varied significantly across the six London CCGs (range from 30.5% to 47.1%). Having a registered mobile number at the GP predicted greater uptake than not having a registered mobile number 43.6% *vs* 36.9% OR=1.31; 95% CI: 1.19–1.44 *P*<0.001). Limiting the analysis to invitees with a registered mobile who did not return their test kit by the eighth week also showed no significant difference between the intervention (*n*=1393, 16.6%) and the control group (*n*=1346; 15.9% OR=1.05; 95% CI: 0.85–1.28–1.52 *P*=0.67).

### The impact on the intervention by invitation status

Although there was no main intervention effect, a test of interaction showed a significant effect of the intervention according to invitation status (*P*=0.02). There was a 5.6 percentage point difference between uptake of the first-time invitees between the intervention (40.5%) and the control groups (34.9% OR=1.29; 95% CI: 1.04–1.58; *P*=0.02) (Table 3). Among those who had been previously invited to the screening programme at least once before this study, there was no significant difference in uptake between the intervention (41.1%) and the control group (40.5% OR=0.98; 95% CI: 0.89–1.08, *P*=0.66). A further exploratory analysis showed no effect of the intervention on uptake when stratified by IMD quintiles, age groups, CCGs, or gender (Supplementary Table S5).

### Mobile phone coverage and delivery of text-messages

Mobile phone coverage was assessed in the screening-eligible population. Coverage varied according to age, gender, IMD score, and CCG (Table 4). Compared with those aged 60–64 years, men and women aged 65–69 years (OR=0.85; 95% CI: 0.77–0.95; *P*=0.004) and 70–74 years (OR=0.67; 95% CI: 0.59–0.75; *P*<0.001) were less likely to have a registered mobile number. The proportion of people with a registered mobile number increased with area-level deprivation, with mobile registration being 53.3% in the most deprived IMD quintile, compared with 42.7% in the least deprived IMD quintile (OR=1.67; 95% CI: 1.39–2.02; *P*<0.001). Mobile registration at GPs also varied by CCG. The largest proportion of individuals with registered mobile phone numbers was observed at Croydon CCG, where coverage was 61.9% (*n*=1109 out of 1791). The lowest was observed in Hammersmith and Fulham CCG, where coverage was 32.2% (*n*=186 out of 577; OR=0.29; 95% CI: 0.24–0.36 *P*<0.001).

Despite differences in GP mobile phone registration, we found no statistically significant difference in the successful delivery of text-messages by participating CCG (*P*=0.08), age group (*P*=0.09), gender (*P*=0.45), IMD quintile (*P*=0.41), or invitation status (*P*=0.07). Among 1393 people in the intervention group with a registered mobile and no returned kit, 73.4% (*n*=1023) successfully received their reminders on the eighth week of their screening episode. Further details of the delivery of text-messages can be found in online Supplementary Materials (Supplementary Table S6).

## Discussion

This trial examined the effectiveness of adding a text-message reminder to the current NHS BCSP through the involvement of primary care. The primary analysis showed that the intervention was not effective at improving uptake of adequate gFOBt screening within six CCGs in London. Likewise, the secondary analysis, limited to individuals who had not returned their test kit within 8 weeks and had a registered mobile number at primary care, indicated that there was no significant difference between the trial arms. However, the subgroup analysis showed that the intervention increased uptake of gFOBt uptake among first-time invitees from 34.9% to 40.1%.

Overall, uptake within the six CCGs (Croydon, Greenwich, Hammersmith and Fulham, Hounslow, Lewisham, West London) was 40.2%, which is consistent with uptake in all of London (40.8%), reported for the first 2.6 million invitations in 2011 (von Wagner *et al*, 2011). The social gradient in uptake in this text-message reminder trial was consistent with the trends in first-time and repeat CRC uptake, specifically with men consistently having lower uptake than women, and people living in the most deprived areas of the six CCGs having poorer uptake rates than those in the least deprived areas (von Wagner *et al*, 2011; Lo *et al*, 2014; Moss *et al*, 2016).

If CRC screening-eligible people complete their gFOBt test kit when they are first invited, it is very likely that they will continue to do so following subsequent invitations (Janda *et al*, 2010; Lo *et al*, 2015b). A previous study on repeat screening participation over two rounds of screening invitations demonstrated that while 86% of previous responders completed their second invitation, only 23% of the previous non-responders returned the test kit (Lo *et al*, 2015a). Hence, the effect of this intervention on the first-time invitees has important implications for the CRC screening uptake, specifically to minimise practical barriers among individuals who have not engaged with screening before their first invitation.

### Strengths and limitations

To our knowledge, this is the first large-scale trial examining the effectiveness of text-message reminders in the context of gFOBT-based CRC screening in a nationally organised programme. The trial was purposefully designed to test a sustainable and a low cost (<£0.05 per text) alternative to postal reminders (second Class UK postage=£0.55), with zero workload to GPs and minimal opportunity costs to the NHS BCSP (Hirst *et al*, 2016). However, there may likely be a one-off investment cost (e.g., change in IT infrastructure) to the NHS BCSP, if text-message reminders were directly sent from the screening programme. Unlike an appointment reminder, the text-message reminders in CRC screening will be limited to non-responders, if applied within NHS BCSP. Unfortunately, possible inaccurate and out-of-date mobile numbers and low proportions of people with registered mobile phone numbers in primary care raise concerns that GP clinical records for mobile phone numbers may not be up-to-date.

It is important to note a number of potential modifications, which could improve the impact of the text-message reminder. In this study, the reminder was sent 8 weeks after the person was mailed their invitation. It is possible that at this point many people would have already misplaced or thrown out their test kit. Future research could test whether sending a text-message reminder earlier in the episode could have more impact on uptake. Relatedly, previous studies that have shown an effect of text-message reminders on CRC and breast screening uptake had informed participants at the time of initial screening invitation that they may receive a text-message reminder (Baker *et al*, 2014; Kerrison *et al*, 2015). Sending a ‘note’ at the start of the trial may act as a ‘primer’ and enhance the impact of a text-message reminder.

## Conclusion

GP-endorsed text-reminders at 8 weeks past the initial invitation of CRC screening did not increase overall uptake in a socioeconomically deprived and ethnically diverse areas in London but their positive impact on first-time invitees is promising and could pay long-term dividends for the effectiveness of the programme.

## Figures and Tables

**Table 1:** Sample characteristics

	**% (*****n***)
Overall	100 (*n*=8269)
Age, years	
60–64	44.5 (3682)
65–69	29.8 (2466)
70–74	25.7 (2121)
Gender	
Male	48.0 (3973)
Female	52.0 (4296)
Index of Multiple Deprivation	
Quintile 1 (least deprived)	8.6 (705)
Quintile 2	14.1 (1157)
Quintile 3	25.0 (2044)
Quintile 4	31.1 (2544)
Quintile 5 (most deprived)	21.1 (1727)
Clinical Commissioning Group	
Croydon	21.7 (1791)
Greenwich	20.7 (1712)
Hammersmith and Fulham	7.0 (577)
Hounslow	19.9 (1645)
Lewisham	16.3 (1349)
West London	14.5 (1195)
Invitation status	
First-time invitees	18.6 (1542)
Repeat invitees	81.4 (6727)

**Table 2:** Intention to treat and secondary analysis

	**Intention-to-treat (** ***N*** **=8269)**			**Secondary analysis (** ***N*** **=2739)**		
	**% uptake (*****N***)	**OR (95% CI)**	***P*** **-value**	**% uptake (*****N***)	**OR (95% CI)**	***P*** **-value**
**Study group**						
Control	39.9 (1648)	Ref.		15.9 (214)	Ref.	
Intervention	40.5 (1674)	1.03 (0.94–1.12)	0.560	16.6 (231)	1.05 (0.85–1.28)	0.670
**Gender**						
Female	42.8 (1837)	Ref.		16.1 (224)	Ref.	
Male	37.4 (1485)	0.80 (0.73–0.88)	0.001	16.4 (221)	0.97 (0.79–1.19)	0.750
**Age, years**						
60–64	38.2 (1407)	Ref.		16.7 (233)	Ref.	
65–69	40.9 (1008)	1.13 (1.00–1.27)	0.050	16.9 (132)	1.14 (0.87–1.49)	0.340
70–74	42.8 (907)	1.26 (1.11–1.43)	0.000	14.2 (80)	0.97 (0.72–1.33)	0.870
**Index of Multiple Deprivation**						
Quintile 1 (least deprived)	51.5 (363)	Ref.		26.4 (178)	Ref.	
Quintile 2	41.9 (485)	0.79 (0.65–0.95)	0.003	23.4 (71)	0.93 (0.59–1.44)	0.740
Quintile 3	43.4 (888)	0.77 (0.64–0.95)	0.000	17.4 (113)	0.62 (0.41–0.92)	0.020
Quintile 4	38.0 (967)	0.61 (0.51–0.73)	0.000	15.0 (137)	0.52 (0.35–0.76)	0.001
Quintile 5 (most deprived)	34.0 (587)	0.53 (0.44–0.64)	0.000	11.0 (72)	0.38 (0.26–0.57)	0.000
**Clinical Commissioning Groups**						
Croydon	47.1 (843)	Ref.		18.5 (129)	Ref.	
Greenwich	43.3 (742)	0.89 (0.77–1.01)	0.080	17.0 (109)	0.93 (0.69–1.25)	0.640
Hammersmith and Fulham	35.9 (207)	0.68 (0.56–0.83)	0.000	14.2 (18)	0.75 (0.44–1.29)	0.290
Hounslow	40.1 (660)	0.77 (0.67–0.89)	0.000	18.6 (73)	0.95 (0.68–1.33)	0.780
Lewisham	37.5 (506)	0.73(0.63–0.85)	0.000	13.8 (77)	0.79 (0.58–1.09)	0.160
West London	30.5 (364)	0.56 (0.47–0.65)	0.000	12.0 (39)	0.65 (0.43–0.96)	0.030
**Invitation status**						
First-time invitee	37.5 (579)	Ref.		18.9 (117)	Ref.	
Repeat invitee	40.8 (2743)	1.01 (0.89–1.16)	0.870	15.5 (328)	0.74 (0.56–0.98)	0.040
**Registered mobile number**						
No	36.9 (1541)	Ref.		NA		
Yes	43.6 (1781)	1.31 (1.19–1.44)	0.000	NA		

Abbreviations: CI=confidence interval; NA=not applicable; OR=odds ratio.

**Table 3:** Impact of the intervention by invitation status

	**Control (** ***n*** **=4135)** a	**Intervention (** ***n*** **=4134)**		
	**% (*****N***)	**% (*****N***)	**OR (95% CI)**	***P*** **-value**
**Invitation status**				
First-time invitee	34.9 (282)	40.5 (297)	1.29 (1.04–1.58)	0.02
Repeat invitee	41.1 (1366)	40.5 (1377)	0.98 (89–1.08)	0.66

Abbreviations: CI=confidence interval; OR=odds ratio.

a Reference group (adjusted for age, gender, Index of Multiple Deprivation, and Clinical Commissioning Group).

**Table 4:** Mobile phone coverage in London among 141 general practices

	**% (*****N***)	**OR (95% CI)**	***P*** **-value**
Overall	49.4 (*N*=4089)		
By gender			
Female	48.4 (*n*=2079)	Ref.	
Male	50.6 (*n*=2010)	1.08 (0.99–1.18)	0.09
By age (years)			
60–64	53.6 (*n*=1972)	Ref.	
65–69	49.2 (*n*=1213)	0.85 (0.77–0.95)	0.004
70–74	42.6 (*n*=904)	0.67 (0.59–0.75)	<0.001
By IMD			
Quintile 1 (least deprived)	42.7 (*n*=301)	Ref.	
Quintile 2	40.7 (*n*=471)	1.24 (1.01–1.51)	0.04
Quintile 3	50.2 (*n*=1026)	1.61 (1.34–1.93)	<0.001
Quintile 4	51.7 (*n*=1316)	1.62 (1.36–1.94)	<0.001
Quintile 5 (most deprived)	53.3 (*n*=920)	1.67 (1.39–2.02)	<0.001
By CCG			
Croydon	61.9 (*n*=1109)	Ref.	
Greenwich	56.1 (*n*=960)	0.79 (0.68–0.90)	0.001
Hammersmith and Fulham	32.2 (*n*=186)	0.29 (0.24–0.36)	<0.001
Hounslow	36.2 (*n*=595)	0.35 (0.30–0.41)	<0.001
Lewisham	58.1 (*n*=784)	0.81 (0.70–0.95)	0.007
West London	38.1 (*n*=455)	0.38 (0.32–0.44)	<0.001

Abbreviations: CCG=Clinical Commissioning Group; CI=confidence interval; IMD=Index of Multiple Deprivation; OR=odds ratio.

## References

[bib1] Baker DW, Brown T, Buchanan DR, Weil J, Balsley K, Ranalli L, Lee JY, Cameron KA, Ferreira MR, Stephens Q, Goldman SN (2014) Comparative effectiveness of a multifaceted intervention to improve adherence to annual colorectal cancer screening in community health centers: a randomized clinical trial. JAMA Intern Med 174(8): 1235–1241. 24934845 10.1001/jamainternmed.2014.2352

[bib2] Baron RC, Rimer BK, Breslow RA, Coates RJ, Kerner J, Melillo S, Habarta N, Kalra GP, Chattopadhyay S, Wilson KM, Lee NC (2008) Client-directed interventions to increase community demand for breast, cervical, and colorectal cancer screening: a systematic review. Am J Prev Med 35(1): S34–S55. 18541187 10.1016/j.amepre.2008.04.002

[bib3] Cancer Research UK (2016)a Bowel Cancer Incidence Statistics Available at http://www.cancerresearchuk.org/health-professional/cancer-statistics/statistics-by-cancer-type/bowel-cancer/incidence last accessed 8 November 2016.

[bib4] Cancer Research UK (2016)b Bowel Cancer Mortality Statistics Available at http://www.cancerresearchuk.org/health-professional/cancer-statistics/statistics-by-cancer-type/bowel-cancer/mortality last accessed 8 November 2016.

[bib5] Camilloni L, Ferroni E, Cendales BJ, Pezzarossi A, Furnari G, Borgia P, Guasticchi G, Rossi PG (2013) Methods to increase participation in organised screening programs: a systematic review. BMC Public Health 13(1): 1. 23663511 10.1186/1471-2458-13-464 PMC3686655

[bib6] Davis TC, Arnold CL, Bennett CL, Wolf MS, Reynolds C, Liu D, Rademaker A (2014) Strategies to improve repeat fecal occult blood testing cancer screening. Cancer Epidemiol Biomarkers Prev 23(1): 134–143. 24192009 10.1158/1055-9965.EPI-13-0795 PMC3894742

[bib7] Gurol–Urganci I, de Jongh T, Vodopivec–Jamsek V, Atun R, Car J (2013) Mobile phone messaging reminders for attendance at healthcare appointments. Cochrane Database Syst Rev (12): CD007458. 24310741 10.1002/14651858.CD007458.pub3 PMC6485985

[bib8] Halloran SP (2009) Bowel cancer screening. Surgery (Oxford) 27(9): 397–400.

[bib9] Hewitson P, Glasziou P, Watson E, Towler B, Irwig L (2008) Cochrane systematic review of colorectal cancer screening using the fecal occult blood test (hemoccult): an update. Am J Gastroenterol 103(6): 1541–1549. 18479499 10.1111/j.1572-0241.2008.01875.x

[bib10] Hirst Y, Kerrison R, Kobayashi LC, Counsell N, Djedovic N, Ruwende J, Stewart M, von Wagner C (2016) Text Reminders in Colorectal Cancer Screening (TRICCS): protocol for a randomised controlled trial. BMC Public Health 16(1): 74. 26809344 10.1186/s12889-016-2733-6 PMC4727285

[bib11] House of Commons (2014) Number of people who were eligible and who participated in the bowel cancer screening programme — England Available at http://www.publications.parliament.uk/pa/cm201314/cmhansrd/cm140401/text/140401w0001.htm#1404026000191 (last accessed 8 November 2016).

[bib12] Icheku V, Arowobusoye N (2015) Evaluation of a service intervention to improve uptake of breast cancer screening in a London Borough with many hard to reach communities. Univ J Public Health 3(2): 92–102.

[bib13] iPlato (2013) iPlato, about us 2013. Available at http://www.iplato.net/about last accessed 9 November 2016.

[bib14] Janda M, Hughes KL, Auster JF, Leggett BA, Newman BM (2010) Repeat participation in colorectal cancer screening utilizing fecal occult blood testing: a community-based project in a rural setting. J Gastroenterol Hepatol 25(10): 1661–1667. 20880176 10.1111/j.1440-1746.2010.06405.x

[bib15] Janz NK, Lakhani I, Vijan S, Hawley ST, Chung LK, Katz SJ (2007) Determinants of colorectal cancer screening use, attempts, and non-use. Prev Med 44(5): 452–458. 17196247 10.1016/j.ypmed.2006.04.004

[bib16] Kaplan WA (2006) Can the ubiquitous power of mobile phones be used to improve health outcomes in developing countries? Global Health 2: 9. 16719925 10.1186/1744-8603-2-9 PMC1524730

[bib17] Kerrison RS, Shukla H, Cunningham D, Oyebode O, Friedman E (2015) Text-message reminders increase uptake of routine breast screening appointments: a randomised controlled trial in a hard-to-reach population. BJC 112(6): 1005–1010. 25668008 10.1038/bjc.2015.36 PMC4366892

[bib18] Levin B, Lieberman DA, McFarland B, Smith RA, Brooks D, Andrews KS, Dash C, Giardiello FM, Glick S, Levin TR, Pickhardt P (2008) Screening and surveillance for the early detection of colorectal cancer and adenomatous polyps, 2008: a joint guideline from the American Cancer Society, the US Multi–Society Task Force on Colorectal Cancer, and the American College of Radiology. CA Cancer J Clin 58(3): 130–160. 18322143 10.3322/CA.2007.0018

[bib19] Lo SH, Halloran S, Snowball J, Seaman H, Wardle J, von Wagner C (2014) Colorectal cancer screening uptake over three biennial invitation rounds in the English bowel cancer screening programme. Gut 64(2): 282–291. 24812001 10.1136/gutjnl-2013-306144 PMC4316922

[bib20] Lo SH, Halloran S, Snowball J, Seaman H, Wardle J, Von Wagner C (2015) aPredictors of repeat participation in the NHS bowel cancer screening programme. BJC 112(1): 199–206. 25429524 10.1038/bjc.2014.569 PMC4453613

[bib21] Lo SH, Waller J, Vrinten C, von Wagner C (2015) Micro actions in colorectal cancer screening participation: a population-based survey study. BMC Cancer 15(1): 438. 26016989 10.1186/s12885-015-1465-9 PMC4446849

[bib22] Matsumoto M, Nishimura T (1998) Mersenne twister: a 623-dimensionally equidistributed uniform pseudo-random number generator. ACM Trans Model Comput Simul 8: 3–30.

[bib23] Moss S, Mathews C, Day TJ, Smith S, Seaman HE, Snowball J, Halloran SP (2016) Increased uptake and improved outcomes of bowel cancer screening with a faecal immunochemical test: results from a pilot study within the national screening programme in England. Gut e-pub ahead of print 7 June 2016; doi: 10.1136/gutjnl-2015-310691. 10.1136/gutjnl-2015-310691 27267903

[bib24] Myers RE, Sifri R, Hyslop T, Rosenthal M, Vernon SW, Cocroft J, Wolf T, Andrel J, Wender R (2007) A randomized controlled trial of the impact of targeted and tailored interventions on colorectal cancer screening. Cancer 110(9): 2083–2091. 17893869 10.1002/cncr.23022

[bib25] Ofcom C (2016) The Communications Market Reports Available at http://stakeholders.ofcom.org.uk/binaries/research/cmr/cmr16/uk/CMR_UK_2016.pdf (last accessed 9 November 2016).

[bib26] Power E, Miles A, von Wagner C, Robb K, Wardle J (2009) Uptake of colorectal cancer screening: system, provider and individual factors and strategies to improve participation. Future Oncol 5(9): 1371–1388. 19903066 10.2217/fon.09.134

[bib27] Scholefield JH, Moss S, Sufi F, Mangham CM, Hardcastle JD (2002) Effect of faecal occult blood screening on mortality from colorectal cancer: results from a randomised controlled trial. Gut 50(6): 840–844. 12010887 10.1136/gut.50.6.840 PMC1773232

[bib28] Segnan N, Patnick J, Von Karsa L (Eds) (2010) European Guidelines for Quality Assurance in Colorectal Cancer Screening and Diagnosis. Office for Official Publications of the European Communities: European Commission.

[bib29] Senore C, Inadomi J, Segnan N, Bellisario C, Hassan C (2015) Optimising colorectal cancer screening acceptance: a review. Gut 64(7): 1158–1177. 26059765 10.1136/gutjnl-2014-308081

[bib30] Sung JJ, Ng SC, Chan FK, Chiu HM, Kim HS, Matsuda T, Ng SS, Lau JY, Zheng S, Adler S, Reddy N (2015) An updated Asia Pacific Consensus Recommendations on colorectal cancer screening. Gut 64(1): 121–132. 24647008 10.1136/gutjnl-2013-306503

[bib31] van Dam L, Korfage IJ, Kuipers EJ, Hol L, van Roon AH, Reijerink JC, van Ballegooijen M, van Leerdam ME (2013) What influences the decision to participate in colorectal cancer screening with faecal occult blood testing and sigmoidoscopy? Eur J Cancer 49(10): 2321–2330. 23571149 10.1016/j.ejca.2013.03.007

[bib32] van Rijn AF, van Rossum LG, Deutekom M, Laheij RJ, Fockens P, Bossuyt PM, Dekker E, Jansen JB (2008) Low priority main reason not to participate in a colorectal cancer screening program with a faecal occult blood test. J Public Health (Oxf) 30(4): 461–465. 18716047 10.1093/pubmed/fdn063

[bib33] von Wagner C, Baio G, Raine R, Snowball J, Morris S, Atkin W, Obichere A, Handley G, Logan RF, Rainbow S, Smith S, Halloran S, Wardle J (2011) Inequalities in participation in an organized national colorectal cancer screening programme: results from the first 2.6 million invitations in England. Int J Epidemiol 40(3): 712–718. 21330344 10.1093/ije/dyr008

[bib34] White B, Power E, Ciurej M, Lo SH, Nash K, Ormiston-Smith N (2015) Piloting the impact of three interventions on guaiac faecal occult blood test uptake within the NHS Bowel Cancer Screening Programme. Biomed Res Int 2015: 928251. 26525423 10.1155/2015/928251 PMC4615211

