# Supplementary material for: Text-message Reminders in Colorectal Cancer Screening (TRICCS): a randomised controlled trial
Source: Br J Cancer. 2017 Apr 25;116(11):1408–14. doi: 10.1038/bjc.2017.117 (PMC5520096; doi:10.1038/bjc.2017.117)
Supplement: Supplementary Table S5 [file bjc2017117x1.docx]

| Table 5 *Tests of interaction* | | | |  |
| --- | --- | --- | --- | --- |
|  | **Control (n=4135)** | **Intervention**  **(n=4134)** | **OR (95% CI)** | **P-value** |
| Gender | | | | |
| Female | 42.0 (916) | 43.5 (921) | Ref |  |
| Male | 37.4 (732) | 37.3 (753) | 0.95 (0.79-1.14) | 0.57 |
| Age | | | | |
| 60-64 | 37.3 (694) | 39.2 (713) | Ref |  |
| 65-69 | 41.6 (510) | 40.2 (498) | 0.86 (0.69-1.06) | 0.18 |
| 70-74 | 42.4 (444) | 43.2 (463) | 0.95 (0.76-1.18) | 0.64 |
| IMD | | | | |
| Quintile 1 | 51.9 (176) | 51.1 (187) | Ref |  |
| Quintile 2 | 39.7 (232) | 44.2 (253) | 1.29 (0.88-1.88) | 0.19 |
| Quintile 3 | 42.6 (432) | 44.3 (456) | 1.11 (0.79-1.59) | 0.54 |
| Quintile 4 | 37.7 (485) | 38.3 (777) | 1.06 (0.75-1.48) | 0.74 |
| Quintile 5 | 35.7 (557) | 32.3 (278) | 0.88 (0.62-1.27) | 0.50 |
| Clinical Commissioning Groups | | | | |
| Croydon | 46.1 (416) | 48.0 (427) | Ref |  |
| Greenwich | 44.2 (387) | 42.4 (355) | 1.04 (0.76-1.42) | 0.81 |
| Hammersmith &Fulham | 36.1 (100) | 35.7 (107) | 0.91 (0.66 -1.24) | 0.54 |
| Hounslow | 37.9 (314) | 42.4 (346) | 0.98 (0.64-1.49) | 0.91 |
| Lewisham | 37.9 (259) | 37.1 (247) | 1.18 (0.86-1.62) | 0.32 |
| West London | 30.2 (172) | 30.7 (192) | 0.94 (0.67-1.32) | 0.73 |
| Invitation Status | | | | |
| *First-time invitees* | 34.9 (282) | 40.5 (297) | 1.30 (1.03-1.64) | 0.02 |
| Repeat Invitees | 41.1 (1366) | 40.5 (1377) | Ref |  |

- All results were adjusted by age, gender, CCG, IMD quintile, invitation status; *p<.05, ***p<.001
