# Supplementary material for: Text-message Reminders in Colorectal Cancer Screening (TRICCS): a randomised controlled trial
Source: Br J Cancer. 2017 Apr 25;116(11):1408–14. doi: 10.1038/bjc.2017.117 (PMC5520096; doi:10.1038/bjc.2017.117)
Supplement: Supplementary Table S6 [file bjc2017117x2.docx]

| Table 6 *Successful Delivery of a text-message* | | | |  |
| --- | --- | --- | --- | --- |
|  | Delivered | Undelivered | χ^2^ | p-value |
| Female | 74.4% (502) | 25.6% (173) | 0.58 | 0.45 |
| Male | 72.6% (521) | 27.4% (197) |  |  |
| Age | | | | |
| 60-64 | 76.1% (521) | 23.9% (164) | 4.85 | 0.09 |
| 65-69 | 70.4% (286) | 29.6% (120) |  |  |
| 70-74 | 71.5% (216) | 28.5% (86) |  |  |
| IMD | | | | |
| Quintile 1 (least deprived) | 79.4% (77) | 20.6% (20) | 3.97 | 0.41 |
| Quintile 2 | 75.5% (117) | 24.5% (38) |  |  |
| Quintile 3 | 74.6% (249) | 25.4% (85) |  |  |
| Quintile 4 | 72.8% (330) | 27.8% (123) |  |  |
| Quintile 5 (most deprived) | 70.4% (238) | 29.6% (100) |  |  |
| Clinical Commissioning Groups | | | | |
| Croydon | 70.3% (253) | 29.7% (107) | 9.71 | 0.08 |
| Greenwich | 72.7% (242) | 27.3% (91) |  |  |
| Hammersmith &Fulham | 84.4% (54) | 15.6% (10) |  |  |
| Hounslow | 79.4% (154) | 20.6% (40) |  |  |
| Lewisham | 71.9% (200) | 28.1% (78) |  |  |
| West London | 73.2% (120) | 26.8% (44) |  |  |
| Invitation Status | | | | |
| *First-time invitees* | 77.7% (220) | 22.3% (63) | 3.67 | 0.07 |
| Repeat Invitees | 72.3% (803) | 27.7 (307) |  |  |
| Total |  |  |  |  |
|  | 73.4% (1023) | 26.6%(370) |  |  |
