# Supplementary material for: Text-message Reminders in Colorectal Cancer Screening (TRICCS): a randomised controlled trial
Source: Br J Cancer. 2017 Apr 25;116(11):1408–14. doi: 10.1038/bjc.2017.117 (PMC5520096; doi:10.1038/bjc.2017.117)
Supplement: Supplementary Table S7 [file bjc2017117x3.docx]

| **Table 7. Univariable Logistic Regression Results for Intention to Treat and Secondary Analysis** | | | | | | |
| --- | --- | --- | --- | --- | --- | --- |
|  | **Intention-to-treat (N=8269)** |  |  | **Secondary Analysis (N=2739)** |  |  |
|  | **% uptake (N)** | **OR (95% CI)** | p-value | **% uptake (N)** | **OR (95% CI)** | p-value |
| Study Group | |  |  |  |  |  |
| Control | 39.9 (1648) | Ref |  | 15.9 (214) | Ref |  |
| Intervention | 40.5 (1674) | 1.03 (.94-1.12) | 0.554 | 16.6 (231) | 1.05 (.86-1.29) | 0.628 |
